# Supplementary material for: De Novo Purine Nucleotide Biosynthesis Pathway Is Required for Development and Pathogenicity in Magnaporthe oryzae
Source: J Fungi (Basel). 2022 Aug 29;8(9):915. doi: 10.3390/jof8090915 (PMC9502316; doi:10.3390/jof8090915)
Supplement: Supplementary file 1 [file jof-08-00915-s001.zip › jof-1808886-supplementary.pdf]

Table S1. Primers used in this study.

Vectors and Primers	Characters or Sequences (5′ to 3′)
MoAde8 upF	GCTAACTGACACTCTAGAGCGTTATCATTGTGTTTGGAGG
MoAde8 upR	TGTTGACCTCCACTAGGTGAAGAAAGTAGGACGT
MoAde8 downF	GCAAAGGAATAGAGTAGATGTTCGACATCACATTGCTC
MoAde8 downR	GGCCAGTGCCAAGCTTCGTTAGGCTGACGGTTGGGATT
MoAde8 upyzF	GGAGTGGGTTGTGGTTAGC
MoAde8 innerF	AACCGCAAGAACGCTTAC
MoAde8 innerR	CGTCTCGGCAATCTCCTT
HPHF	TAGTGGAGGTCAACAATGAATG
HPHR	CATCTACTCTATTCCTTTGCC
MoAde8 GFPF	ATCAATCACAATGGCCATGGCAGCTCCCATCAGGAT
MoAde8 GFPR	CGCCCTTGCTCACCATCCTCTTCTTCGTCTCGGCAATC
MoAde5 upF	GCTAACTGACACTCTAGAAACGGCAGCTGAAGATGG
MoAde5 upR	TGTTGACCTCCACTATTGACTAGGGTCCTACGG
MoAde5 downF	GCAAAGGAATAGAGTAGATGTAGAGCTGCATTTCTTCAC
MoAde5 downR	GGCCAGTGCCAAGCTTATGCAAGCGCAAGCCAAG
MoAde5 upyzF	TTTGATGGCGATGGCGGAG
MoAde5 innerF	CGGTATCGGTATGGCTATC
MoAde5 innerR	TGGTCACTACAGGTAAGGT
MoAde5 GFPF	ATCAATCACAATGGCCATGGAGTCTCTCCGTGTT
MoAde5 GFPR	CGCCCTTGCTCACCATAGCAGCCCAGGCCTCCAAGTTT
MoAde6 upF	GCTAACTGACACTCTAGATCATGTCGTTTGTCGATCCTC
MoAde6 upR	TGTTGACCTCCACTACAAGGAAAATTTGGAGGG
MoAde6 downF	GCAAAGGAATAGAGTAGATGGGTATACTGTTTACATCGGTCG
MoAde6 downR	GGCCAGTGCCAAGCTTTTGTCTCTCCTCAGGTCCTTTT
MoAde6 upyzF	GTTGAAAAAGGCCTATGGC
MoAde6 innerF	ATTTCGCAGGTCCTCCAATGAG
MoAde6 innerR	CGAGCCAGGGTTTCGGTCATTC
MoAde6 GFPF	ATCAATCACAATGGCCATGCCGCACGAGGTTCTGGTT
MoAde6 GFPR	CGCCCTTGCTCACCATACCAACCCACTTTCTCGCGT
MoAde12 upF	GCTAACTGACACTCTAGACGTTGGGACGTGACGATGAT
MoAde12 upR	TGTTGACCTCCACTAGCTTTGTGTGGGTTCCTC
MoAde12 downF	GCAAAGGAATAGAGTAGATGCCCTATTACCGTGAAACTTG
MoAde12 downR	GGCCAGTGCCAAGCTTATCCCCTTACGGCGCAGCT
MoAde12 upyzF	GCAAATCAAGGAGAAGCT
MoAde12 innerF	TGAGTCTTACCCTGTCTC
MoAde12 innerR	ATAGGATACGTACAGCAGAGCG
MoAde12 GFPF	ATCAATCACAATGGCCATGGCTACCATTATTCTGGG
MoAde12 GFPR	CGCCCTTGCTCACCATTCGATATATCATGTCCTCGCG
MoAde4 upF	GCTAACTGACACTCTAGAGCAGAGCAGGCTCAGGAATT
MoAde4 upR	TGTTGACCTCCACTAAAAGGCGACGCCGATCTT
MoAde4 downF:	GGAATAGAGTAGATGGTGTTGAATGAGGTGATGGGAC
MoAde4 downR:	GGCCAGTGCCAAGCTTCCGCTTACGCCAGTTATTT
MoAde4 upyzF:	ATGTGGGGTTTGCTGAAGCT

MoAde4 innerF:	TTGTAAGGCCATGACCCCTCC
MoAde4 innerR:	GATTCCCATGAAGCCCGGTAGG
YZ-tubulinF	CGATGGCTTTCGCCTCAAGTCGTCC
YZ-tubulinR	CACTCACAAGATGGCAGAGCAGGTCAGGTA
HPHlongR	GATAATAATGTCCTCGTTCC
MoAde8 ADF:	GGAGGCCAGTGAATTCATGGCAGCTCCCATCAGGATATC
MoAde8 ADR:	CGAGCTCGATGGATCCCCTCTTCTTCGTCTCGGCAAT
MoAde1 ADF:	GGAGGCCAGTGAATTCATGGCCGCATCAGAAGCCGTCAC
MoAde1 ADR:	CGAGCTCGATGGATCCCGGAAACCCATCCTTCAGT
MoAde2 ADF:	GGAGGCCAGTGAATTCATGTCTCAAGAAACAGTTGTAGG
MoAde2 ADR:	CGAGCTCGATGGATCCCTTCTTACCCATTTTGGCC
MoAde3 ADF:	GGAGGCCAGTGAATTCATGATAGCTGATGGAAAAC
MoAde3 ADR:	CGAGCTCGATGGATCCAAACAGACCGTCAATCTCGC
MoAde4 ADF:	GGAGGCCAGTGAATTCATGTGTGGTGTATCCGCGCTCC
MoAde4 ADR:	CGAGCTCGATGGATCCAGGTTTGGTTGCGAAGTT
MoAde5 ADF:	GGAGGCCAGTGAATTCATGGAGTCTCTCCGTGTTCTT
MoAde5 ADR:	CGAGCTCGATGGATCCAGCAGCCCAGGCCTCCAAGTTT
MoAde6 ADF:	GGAGGCCAGTGAATTCATGCCGCACGAGGTTCTGGTTG
MoAde6 ADR:	CGAGCTCGATGGATCCACCAACCCACTTTCTCGCG
MoAde12 ADF:	GGAGGCCAGTGAATTCATGGCTACCATTATTCTGGGATC
MoAde12 ADR:	CGAGCTCGATGGATCCTCGATATATCATGTCCTCG
MoAde13 ADF:	GGAGGCCAGTGAATTCATGCCTGACTATGATAGCT
MoAde13 ADR:	CGAGCTCGATGGATCCTACAGAAAGCTCCGCGGTGGCT
qPCR-*MoALB1*-F	ACTAAACGAGCGGTATCATGC
qPCR-*MoALB1*-R	GGTAGGTTTTGTCATGCTGTG
qPCR-*MoRSY1*-F	CGACTCCAAGGACTGGGATA
qPCR-*MoRSY1*-R	GTCCTCGGACACCTTCTCC
qPCR-*MoBUF1*-F	ACGCCGTCTACTCAGGATCA
qPCR-*MoBUF1*-R	TCTCGCCGTTTGGAATGTAT
MoAde4 FlagF	CAATCACAATGGCCGGATCCATGTGTGGTGTATCCGCGCTCCTT
MoAde4 FlagR	TGGTCCTTGTAGTCCCCGGGAGGTTTGGTTGCGAAGTTGTGAAG
MoFkbp12 GFPF	ATCAATCACAATGGCCATGGGTGTTACAAAGACC
MoFkbp12 GFPR	CGCCCTTGCTCACCATTTTGATTCCCTTCAGGTAG
MoFkbp12BDF	CATGGAGGCCGAATTCATGGGTGTTACAAAGACC
MoFkbp12BDR	GCAGGTCGACGGATCCTTTGATTCCCTTCAGGTAGAC

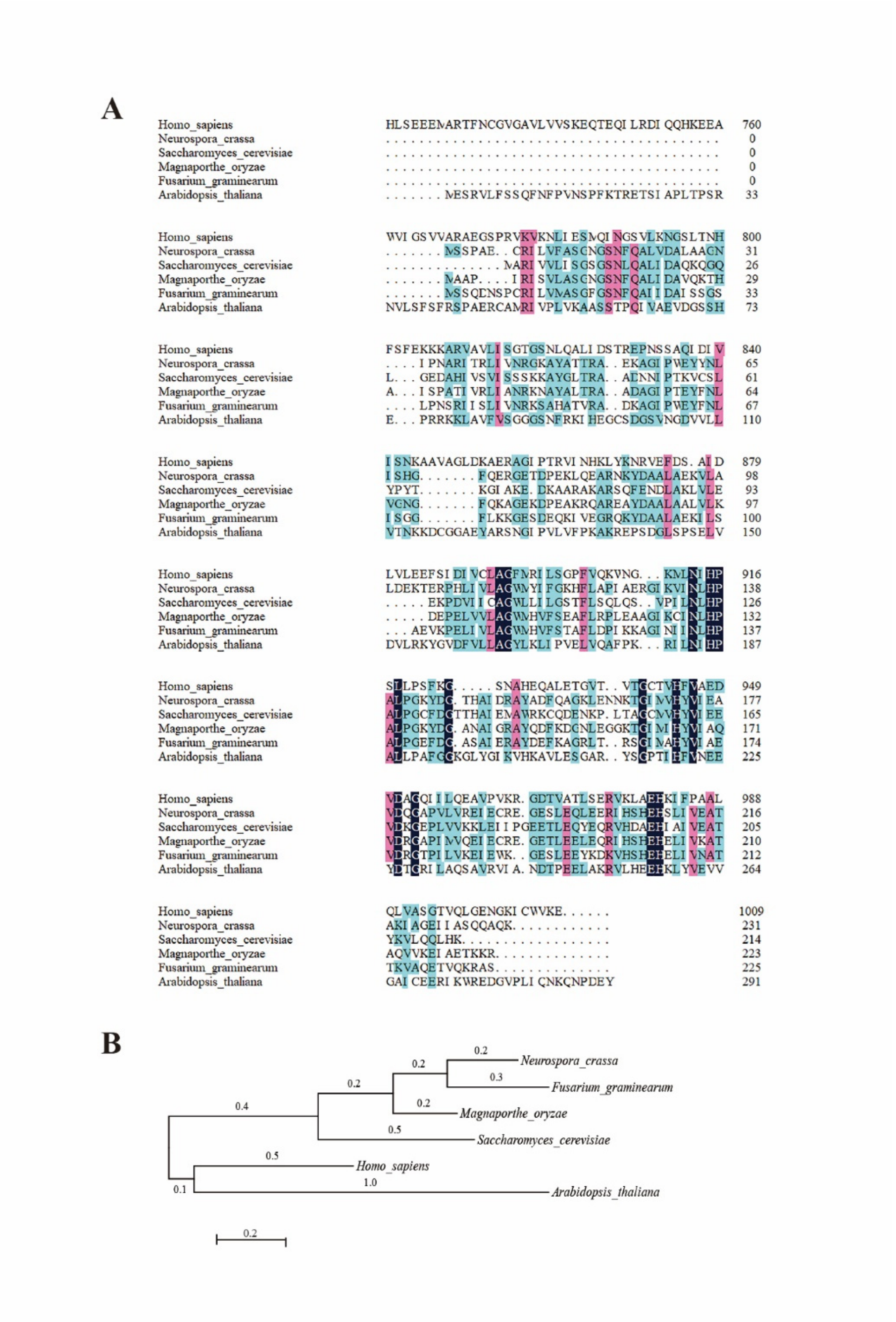

Figure S1. Sequence alignment of Ade8 in different organisms. (**A**)Amino acid sequences of Ade8 in *M. oryzae*, *Homo sapiens*, *Neurospora crassa*, *Saccharomyces cerevisiae*, *Fusarium graminearum*, and *Arabidopsis thaliana* were aligned by DNAMAN8. Amino acid sequences were obtained by querying the UniProt (https://www.uniprot.org/). (**B**) Phylogenetic tree of GART in different species was constructed using MEGA 6.

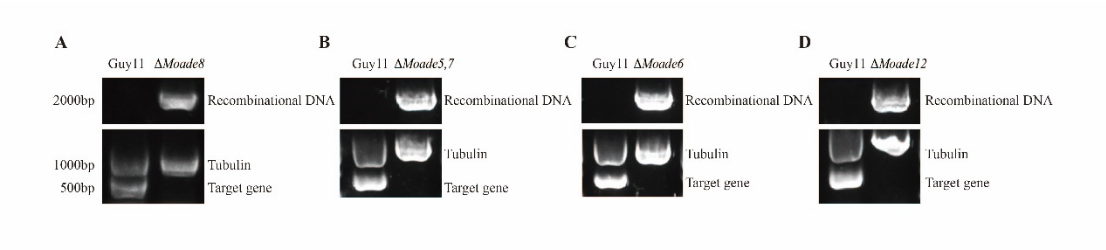

Figure S2. Deletion of DNPB pathway genes. (**A–D**) Deletion of *MoADE8*, *MoADE5,7*, *MoADE6*, and *MoADE12* was verified by the PCR assay.

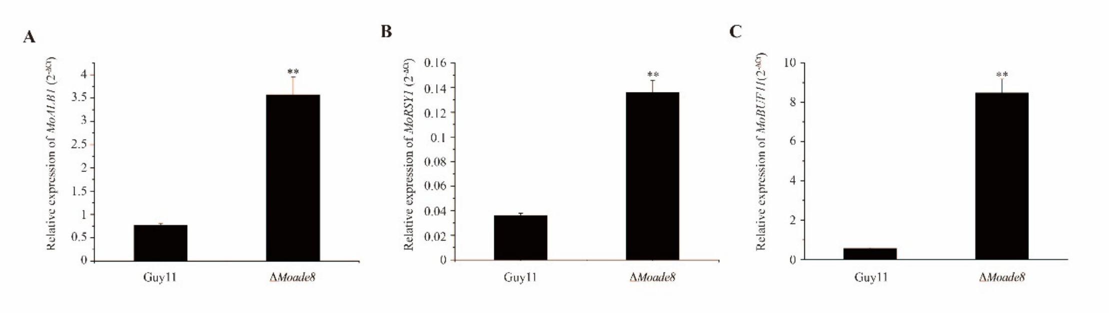

Figure S3. The expression levels of genes related to melanin production. Total RNA was extracted from both the Guy11 and Δ*Moade8* mycelium. The qPCR assay was conducted to show the expression level of *MoALB1* (**A**), *MoRSY1* (**B**), and *MoBUF1* (**C**) in Guy11 and Δ*Moade8*. Asterisks are used to mark significant differences (**$P < 0.01$).

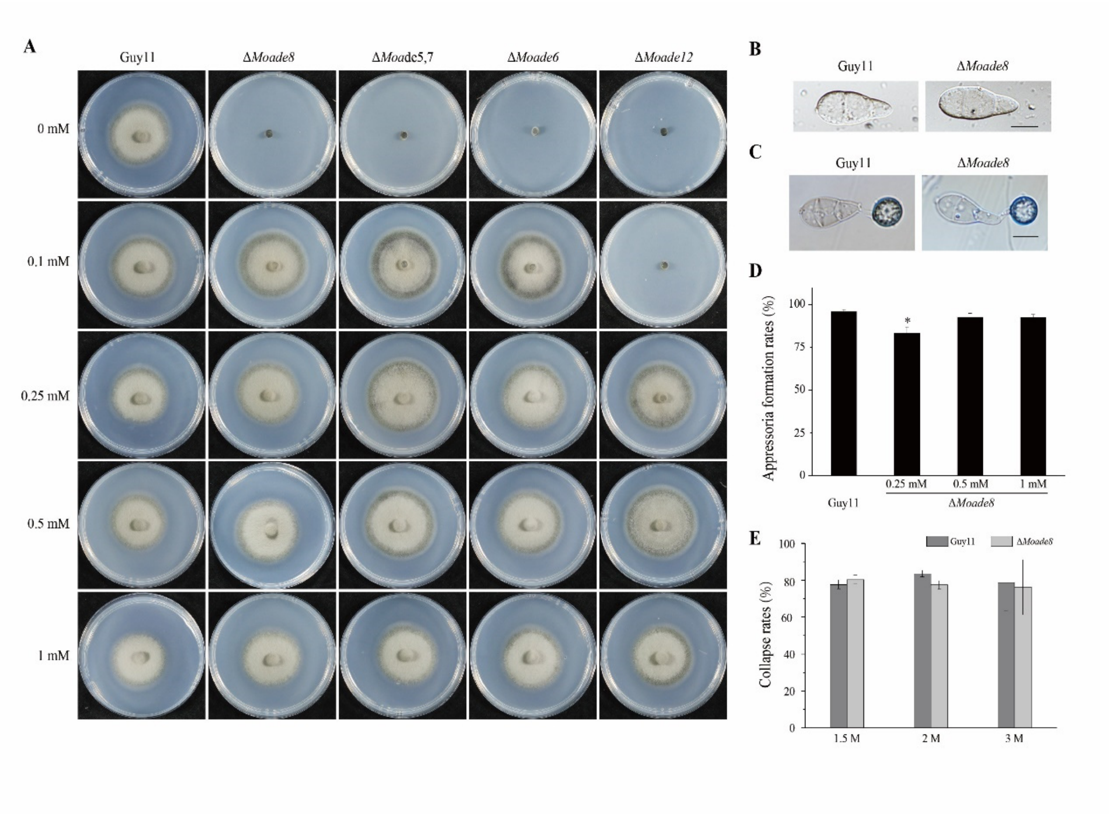

Figure S4. Exogenous adenine rescued the defects of Δ*Moade8*. (**A**) Growth of Guy11,Δ*Moade8*, Δ*Moade5,7*, Δ*Moade6* and Δ*Moade12* on MM medium supplemented with 0 mM, 0.1 mM, 0.25 mM, 0.5 mM and 1 mM adenine for 8 d. (**B**) Conidia morphology of Guy11 and Δ*Moade8*. Scale bar, 10 μm. (**C**) Appressorium morphology of Guy11 and Δ*Moade8*. Scale bar, 10 μm. (**D**) Appressoria formation rates of Guy11 and Δ*Moade8*. Conidia were collected from colonies grown on CM containing 0.25 mM, 0.5 mM, 1 mM adenine. For each strain, 100 conidias were analyzed. Asterisks are used to mark significant differences (*$P < 0.05$). (**E**) Collapse rates of Guy11 and Δ*Moade8* treated with different concentrations of glycerol.

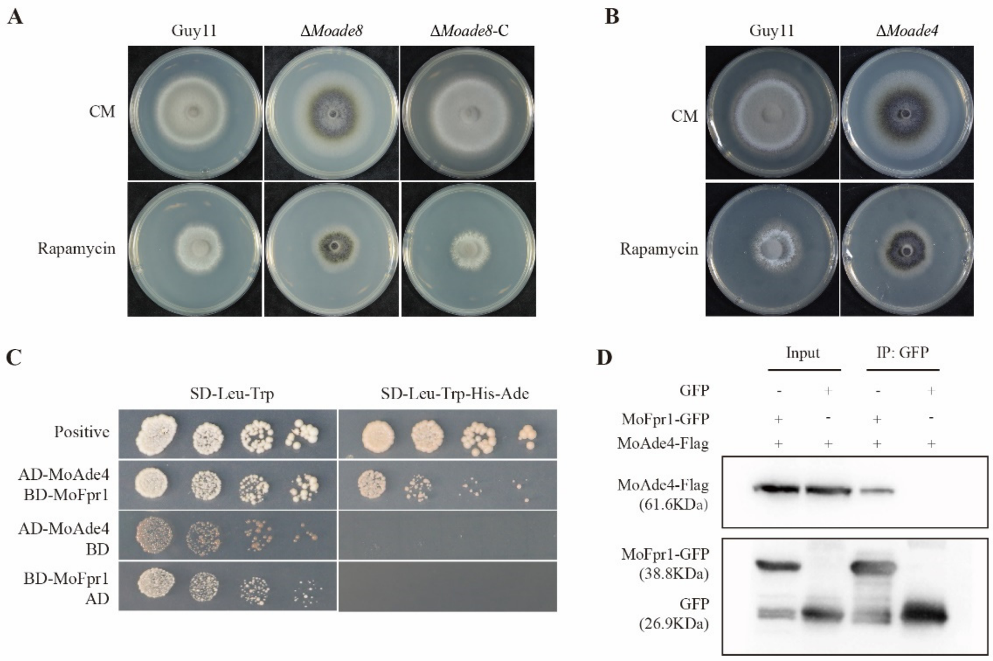

Figure S5. Association between the TOR pathway and DNPB pathway. (**A**) Growth of Guy11, Δ*Moade8* and Δ*Moade8*-C on CM and CM supplemented with 50 nM rapamycin. Photos were taken at 8 dpi. (**B**) Growth of Guy11, Δ*Moade4* on CM and CM supplemented with 50 nM rapamycin. Photos were taken at 8 dpi. (**C**) Yeast two-hybrid assay was conducted to examine the interaction between MoAde4 and MoFpr1. pGBKT7-53 and pGADT7-T were used as the positive controls. (**D**) The co-IP assay was conducted on the strain co-expressed the GFP-fused MoFpr1 and 3×FLAG-fused MoAde4 to verify the interaction of MoFpr12 and MoAde4 *in vivo*. The strain co-expressed GFP and 3×FLAG-fused MoAde4 was used as the control.
